# Exploring a comprehensive knowledge map for bridge management research: a Delphi-enhanced scientometric analysis

**DOI:** 10.1038/s41598-026-51462-6

**Published:** 2026-05-07

**Authors:** Peng Peng, Zuocai Wang, Peng Cui, Hongzhe Yue, Junfeng Yao, Sainan Lyu

**Affiliations:** 1 School of Civil Engineering, Hefei University of Technology, Hefei, 230009 China; 2 School of Civil Engineering, Nanjing Forestry University, Nanjing, 210037 China; 3 College of Civil Engineering, Anhui Jianzhu University, Hefei, 230022 China; 4 Department of Applied Physics and Electronics, Umeå University, 90187 Umeå, Sweden; 5 School of Civil Engineering, Southeast University, Nanjing, 211189 China; 6 School of Management, Hefei University of Technology, Hefei, 230009 Anhui China; 7 School of Economics and Management, Anhui Jianzhu University, Hefei, 230022 China

**Keywords:** Bridge management, Scientometric analysis, Bridge engineering, Engineering management, CiteSpace, Bridge management systems, Engineering, Mathematics and computing

## Abstract

Bridge infrastructure worldwide is aging, while demands for safety, sustainability, and cost-efficiency intensify challenges in bridge management (BM). This study develops a comprehensive knowledge map of BM through scientometric analysis, clarifying its intellectual structure, technological frontiers, and emerging themes. Using CiteSpace, 1582 peer-reviewed articles (2003–2024) from the Web of Science Core Collection were analyzed, with keywords and gaps refined via a Delphi process involving five senior experts. Scientometric techniques—including co-citation, cluster mapping, citation bursts, and keyword time-zone analysis—were employed to trace field evolution. Results reveal three stages: exploratory (2003–2009), steady growth (2010–2016), and digital transformation (2017–2024). Ten thematic clusters emerged, spanning intelligent management, hybrid decision-making, inspection technologies, and life-cycle sustainability. Research hotspots highlight digital twins, machine learning, condition assessment, and structural health monitoring. Five evidence-informed future priorities were identified: integrated AI-driven frameworks, hybrid decision models, network-level inspection planning, sustainability-oriented life-cycle assessment, and robust resilience modeling. Co-authorship and institutional collaboration networks further reveal global hubs shaping BM knowledge exchange. This study provides an updated mapping of BM, offering a structured agenda that links quantitative evidence with practical directions for advancing intelligent, sustainable, and risk-resilient BM.

**Supplementary Information:**

The online version contains supplementary material available at 10.1038/s41598-026-51462-6.

## Introduction

Bridge infrastructure plays a vital role in enabling connectivity, economic activity, and disaster resilience^1–3^. However, aging structures, increasing load demands, budget constraints, and extreme climate events have intensified the complexity of bridge management (BM) in both developed and developing contexts^4^. BM refers to the integrated lifecycle coordination of inspection, assessment, maintenance, and rehabilitation activities across bridge systems^5^. In contrast, bridge management systems (BMS) are the digital or analytical platforms that support this process through data integration, decision algorithms, and visual tools^6^. Throughout this paper, “BM” denotes the management process, whereas “BMS” denotes the software/system platform that supports BM tasks.

In recent years, BM has seen rapid technological convergence, especially through the incorporation of building information modeling (BIM)^4,7^, structural health monitoring (SHM)^8^, artificial intelligence (AI)^9^, and digital twin (DT) technologies^5^. These advances offer new opportunities for data-driven, lifecycle-oriented, and resilient BM strategies. Yet, the field remains fragmented in thematic focus, methodological frameworks, and terminology, limiting effective knowledge transfer and interdisciplinary collaboration^10–12^.

Several literature reviews have attempted to summarize the state of bridge management (BM) research from varying perspectives. For example, Ahmad et al.^13^ focused on predictive modeling and BIM implementation, while Brighenti et al.^14^ reviewed intelligent BMS design approaches. Dayan et al.^4^ examined information modeling in BM, and Milić & Bleiziffer (2024) discussed sustainability and lifecycle assessment practices^15^. However, these studies remain qualitative, technology-specific, or narrow in disciplinary scope. As summarized in Table 1, existing studies typically span limited timeframes, lack quantitative scientometric analysis, and seldom explore the full thematic evolution of BM research. They offer valuable contributions, but they still call for a multi-decade, data-driven knowledge map that captures the field’s intellectual structure, research maturity, and emerging challenges. This highlights the need for a longitudinal scientometric study to uncover how BM research has evolved and where its most critical gaps lie.

**Table 1:** Comparison of existing BM review studies.

No	References	Scientometric analysis	Number (range)	Research focus	Main results
1	^5^	No	183 2010–2020	Investigating the current progress of BMS in the asset management for bridges using digital models such as BIM and digital twins	This paper provides a summary of research progress in the BM field, especially for asset management of bridges from 2010 to 2020
2	^4^	No	78 2010–2020	Verifying different aspects of using information modeling in BM	This study indicates the concentration of BM researchers on information modeling is on new inspection, test methods and maintenance optimization with a particular focus on concrete structures
3	^15^	No	Not mentioned	Exploring the current challenges facing bridge owners and managers as a result of anthropogenic and natural climate changes	This article discusses methodologies for planning under uncertainty, including stochastic and robust optimization methods on BM under climate change
4	^12^	No	Not mentioned	Investigating the emerging information technologies to facilitate enablers and overcome barriers for more intelligent BM	This paper summarizes the business background and big data characteristics of BM, and proposes a novel BigKE-based intelligent bridge management and maintenance framework
5	^9^	No	81 2019–2024	Exploring the use of artificial intelligence in structural health management of bridges	The paper presents a systematic review about the use of artificial intelligence in the field of structural health management of existing bridges
6	^6^	No	Not mentioned	Introducing the history of bridge engineering and the management of bridges	This paper reviews the development of BM and BMS from the history of human innovation and postulates where BM might be heading in the future
7	^13^	No	26 2010–2024	Understanding the importance of using BIM in bridge projects by investigating the role of its proper implementation to avoid and mitigate risks	This study reveals the detailed ways in which BIM contributes to the whole lifespan of a bridge, including exact planning to prevent claims and delays as well as the careful selection of ecologically friendly materials
8	This study	Yes	1582 Not limited	Identifying the development trends of BM: recognizing research progress, evolution, research spotlight, research gaps and future trends	This study unveils the underlying knowledge structure while identifying evolutionary patterns and emerging trends in BM research in a visual, objective, and comprehensive manner. Identified ten clusters representing BM development are highlighted. Five gaps and future directions in BM are proposed

Such a map is not only academically valuable but strategically necessary. In fields with accelerating complexity, scientometric mapping enables evidence-based navigation of research landscapes, revealing intellectual turning points, research gaps, and emerging paradigms^16,17^. It also supports agenda-setting across research, education, and policy domains^18^. For BM specifically, a knowledge map helps scholars consolidate scattered findings, guides infrastructure agencies in identifying priority areas, and aligns innovation efforts with urgent societal needs such as climate adaptation, risk resilience, and sustainable asset lifecycle planning^19–21^.

Despite this relevance, scientometric evidence specific to BM remains relatively limited in providing an integrated, longitudinal view of the field. Existing mapping efforts often focus on individual technologies (e.g., BIM or DT), provide limited methodological transparency, or stop short of linking structural clusters to practice-relevant implications. Accordingly, there is a need for a longitudinal, theory-informed, and practice-oriented knowledge map that traces how BM has evolved, where it stands, and which directions are most promising.

This paper addresses this need through a scientometric analysis of 1582 peer-reviewed articles (2003–2024) from the Web of Science Core Collection. It integrates Delphi-based keyword refinement with clustering, co-citation, and burst analyses using CiteSpace, providing a structured basis for both theoretical synthesis and practice-oriented insights in BM. Compared with studies that rely solely on researcher-defined search strategies and interpretation, a Delphi-based consensus process is incorporated to support keyword refinement and to cross-check the interpretation of major patterns. This approach is intended to improve thematic coverage and contextual consistency, particularly in the multidisciplinary BM field. Beyond conventional scientometric mapping, the study further interprets representative literature within key clusters to connect quantitative patterns with managerial and engineering implications.

To guide the analysis, this study explores the following three research questions (*RQ*):

- *RQ*1: How has the volume and thematic focus of bridge management (BM) research evolved from 2003 to 2024 in the retrieved dataset, and what trends can be identified in the temporal distribution of scholarly output?
- *RQ*2: What are the principal research clusters, core publications, and technological keywords that define the intellectual structure of BM research? How do international and institutional collaborations shape the production and dissemination of BM knowledge?
- *RQ*3: Which underexplored research areas and methodological gaps emerge from the scientometric analysis, and how can they inform the development of future intelligent, sustainable BM?

## Research methodology

**Fig. 1:**
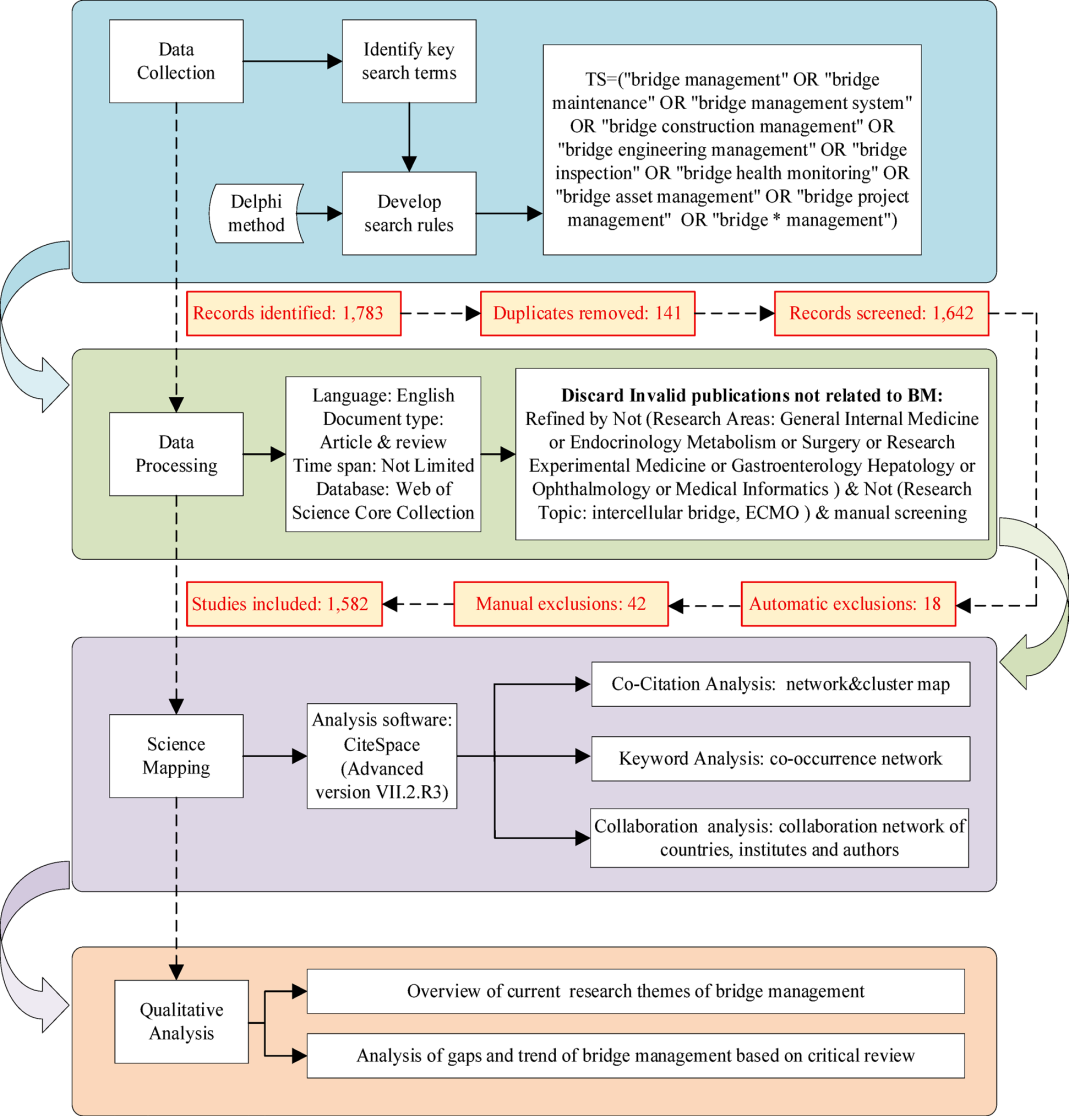
The outline of the research design.

To explore the knowledge structure and development trajectory of BM research, this study adopts a scientometric analysis framework enhanced by Delphi-based keyword refinement and expert interpretation cross-checking. This method enables the objective, quantitative mapping of a discipline’s intellectual structure and evolution over time^19–21^. The research design follows the widely accepted science-mapping framework proposed by Börner^18^, involving 4 phases: tool selection, data collection, visualization, and interpretation. The overall methodological process is shown in Fig. 1.

### Tool selection and rationale

To overcome the limitations of manual reviews, scientometric tools like CiteSpace have been widely adopted^16,17^. These tools enable researchers to detect hidden patterns and emerging trends by integrating quantitative analysis with visualizations, deepening understanding of evolving knowledge domains^20^.

Among various scientometric tools such as VOSviewer, BibExcel, CoPalRed, Sci2, and HistCite, CiteSpace 6.2.R3 was selected due to its comprehensive capabilities in co-citation network generation, keyword clustering, burst detection, and timeline mapping^22^. Compared to other tools, CiteSpace offers superior interpretability and modular cluster analysis using log-likelihood ratio (LLR) and silhouette scores to ensure meaningful grouping^23,24^. For instance, in contrast to VOSviewer, which excels in co-authorship or term mapping but lacks burst detection, CiteSpace provides more robust support for temporal evolution and frontier tracking, key requirements in this study. CiteSpace is also natively compatible with the Web of Science data format, ensuring metadata integrity.

### Keyword strategy via Delphi method

To ensure both breadth and precision of the literature retrieval, we employed a three-round Delphi procedure to refine the topic-search terms. Five senior experts (coded as E1–E5) were recruited via purposive sampling based on predefined eligibility criteria: (1) ≥ 20 years of research or professional experience related to bridge management (BM) (e.g., inspection, maintenance, condition assessment, deterioration modelling, intervention planning, asset management, and BMS decision-support); (2) demonstrated domain expertise evidenced by peer-reviewed publications and/or leadership roles in BM-related projects; (3) familiarity with emerging BM-related digital technologies (e.g., SHM, BIM, digital twins) and their practical applications; and (4) willingness to participate in all three iterative rounds. To balance academic rigor and engineering applicability, the panel included experts from both academia and practice, and their background information is summarised in Supplementary Table S1. In accordance with research ethics and confidentiality requirements, the experts were anonymised and only non-identifiable background information relevant to the study was reported.

Specifically, Panel composition and background are as follows: the Delphi panel comprised five senior experts (E1–E5), including three professors (academia) and two senior engineers (practice). Their ages ranged from 49 to 61 years (mean = 55.2), and their BM-related professional experience ranged from 22 to 36 years (mean = 30.4). Highest degrees included Doctor (n = 1), Master (n = 3), and Bachelor (n = 1) (Supplementary Table S1). The experts served as independent advisors for keyword refinement and interpretation cross-checking, and were not involved in the quantitative CiteSpace computations.

In Round 1, experts independently proposed candidate keywords based on their knowledge of BM research and practice. In Round 2, overlapping, redundant, and ambiguous terms were consolidated through iterative feedback. In Round 3, each candidate term was rated on a 5-point Likert scale (1 = not relevant, 5 = highly relevant). Consensus was assessed using the median and interquartile range (IQR); a keyword was retained when the median score was ≥ 4 and the IQR was ≤ 1, otherwise it was revised and re-evaluated in the next round. To make the Delphi contribution transparent, Supplementary Table S5 reports the round-by-round evolution of the candidate vocabulary, including the initial terms proposed in Round 1, the consolidation or deletion decisions made in Round 2, and the final consensus outcomes used in Round 3.

The final consensus set of keywords was then used to construct the Web of Science Topic Search (TS) query^20,25,26^. For reproducibility, the full rerunnable query is reported here: TS = (“bridge management” OR “bridge maintenance” OR “bridge management system” OR “bridge construction management” OR “bridge engineering management” OR “bridge inspection” OR “bridge health monitoring” OR “bridge asset management” OR “bridge project management” OR “bridge * management”). The wildcard “*” was used to capture lexical variations and reduce omission. Figure 1 summarizes the overall retrieval and screening workflow, whereas the full query is reported here to enable direct replication. In this study, the Delphi exercise was used to (1) refine the topic-search vocabulary for retrieval completeness and precision and (2) cross-check the interpretation of major scientometric patterns; it was not used for statistical inference.

### PRISMA-based screening process

This literature retrieval and screening process followed the PRISMA 2020 guidelines^27^ for transparent reporting of systematic reviews. As illustrated in Fig. 1, the workflow comprised four stages: identification, de-duplication, relevance screening, and inclusion. A total of 1783 records were initially identified from the Web of Science Core Collection. Zotero-based de-duplication removed 141 duplicate records, leaving 1642 unique records for further screening. These unique records then underwent a two-step relevance-control procedure. First, 18 clearly irrelevant biomedical records were automatically excluded through the dual topic/discipline filter. Second, the remaining 1624 records were manually screened by three PhD researchers based on titles, abstracts, and conclusions, leading to the exclusion of a further 42 records. After these post-de-duplication exclusions, 1582 eligible publications were retained for the scientometric analysis. The numerical accounting reported in Fig. 1 should therefore be read as: 1783 identified → 141 duplicates removed → 1642 screened → 18 automatically excluded → 1624 manually screened → 42 manually excluded → 1582 included.

### Data source and cleaning strategy

This study relies exclusively on the Web of Science (WoS) Core Collection due to three key advantages^28^: (1) Curatorial Rigor: WoS applies strict peer-reviewed standards, ensuring high academic quality^29^. (2) Integrated Citation Structure: WoS supports complete co-citation analysis ideal for knowledge mapping^23^. (3) Tool Compatibility: CiteSpace directly supports WoS data formats, reducing formatting errors.

**Fig. 2:**
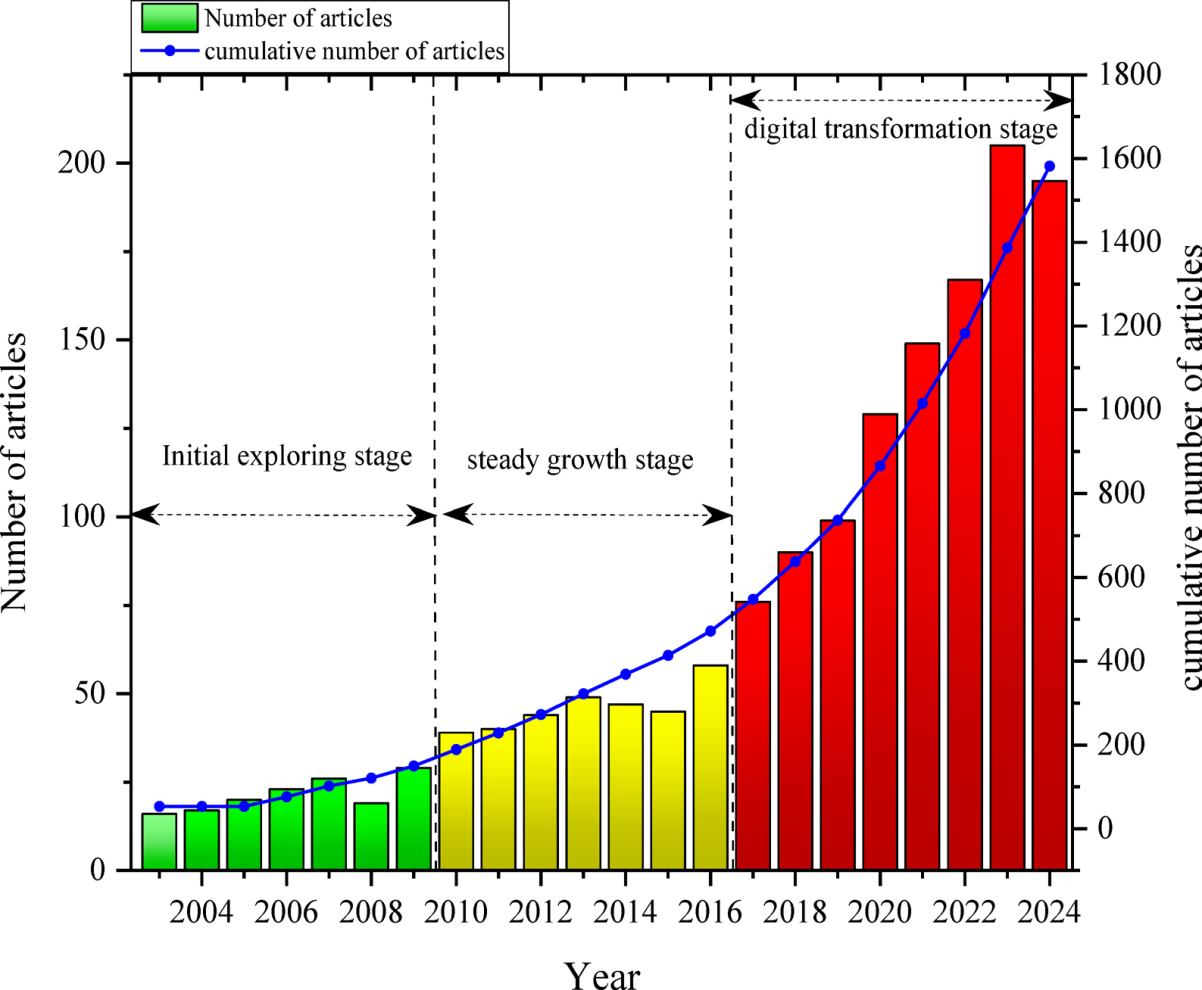
Annual articles number statistics.

A total of 1783 publications were initially retrieved without a time span limit and automated de-duplication was conducted by Zotero. To avoid contamination from irrelevant fields, particularly biomedical literature containing the keywords “intercellular bridge” or “ECMO”, a dual exclusion filter was applied:

1. Exclude research areas: General Internal Medicine, Endocrinology Metabolism, Surgery, Research Experimental Medicine, Gastroenterology Hepatology, Ophthalmology, Medical Informatics; Exclude topic terms: “ECMO”, “intercellular bridge”.
2. After this filtering, manual screening was conducted in three stages by three PhD candidates specializing in BM. Titles, abstracts, and conclusions were assessed to confirm topic relevance. Articles not related to BM were removed according to the criteria summarized in Fig. 1 and detailed in the next section, yielding 1582 valid records from 2003 to 2024 (Fig. 2).

A total of 60 records were excluded after de-duplication through the combined relevance-control procedure. Of these, 18 records were removed automatically by the predefined dual filter, and a further 42 records were excluded during manual screening. These exclusions refer only to post-de-duplication relevance control and do not include the 141 duplicate records removed by Zotero. For example, records containing “bridge management” in biomedical contexts such as “intercellular bridge signaling” were eliminated.

The search was completed on December 20, 2024. In the retrieved WoS sample used in this study, BM-related publications appear from 2003 onward. This observation pertains to the bibliographic dataset analyzed here rather than to the historical origin of BM itself, and it may reflect a combination of database coverage, indexing practices, and search-scope characteristics rather than a definitive beginning of the field. Accordingly, the three-stage periodization adopted in this study should be interpreted as an analytical division of the retrieved literature rather than as a complete historical account of BM development. Following Price’s science growth theory^30^ and the publication pattern observed in the dataset, BM research is categorized here into three developmental stages: the exploratory stage (2003–2009), the steady growth stage (2010–2016), and the digital transformation stage (2017–2024).

### Eligibility criteria for study selection

To ensure reproducibility, we applied explicit inclusion and exclusion criteria. Records were included if they met all of the following: (1) indexed in the Web of Science (WoS) Core Collection; (2) document type limited to peer-reviewed journal publications (Article and Review); (3) written in English; (4) published between 2003 and 2024 (inclusive); and (5) primarily focused on bridge management in civil infrastructure (including inspection, maintenance, condition assessment, deterioration modelling, intervention/maintenance planning, asset management, and BMS-related decision support). Records were excluded if they: (1) belonged to non-civil-infrastructure contexts despite the term “bridge” (biomedical topics like “ECMO” and “intercellular bridge”); (2) were non-peer-reviewed document types (editorial material, meeting abstracts, letters, book chapters); or (iii) lacked sufficient bibliographic metadata (title/abstract/keywords) for relevance judgement and CiteSpace parsing.

### CiteSpace configuration parameters

CiteSpace analysis was performed with the following configuration. (1)Time slicing: 2003–2024 (1-year intervals) (2) Top N: 50 most cited references per slice (3) Node types: Reference co-citation, keyword co-occurrence, clustering (4) Pruning methods: Pathfinder and Minimum Spanning Tree (MST) (5) Quality metrics: Modularity Q for inter-cluster separation and average silhouette for intra-cluster cohesion^20,21^.

## Findings

### Document co-citation analysis

**Fig. 3:**
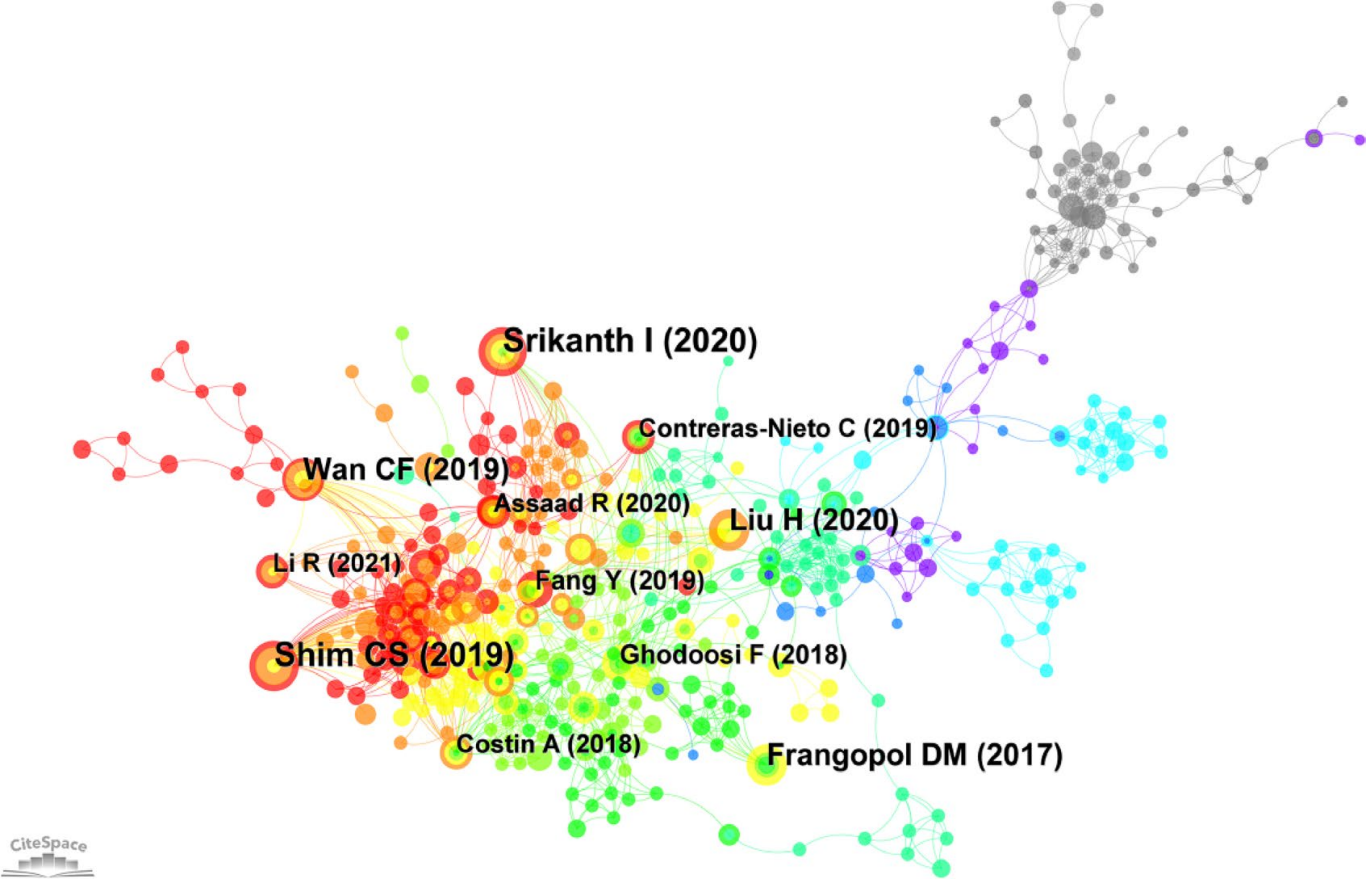
Document co-citation network of bridge management studies.

Based on the development of the knowledge domain, document co-citation analysis was performed on the BM core dataset using CiteSpace. The timeline was divided into annual slices, analyzing the top 50 cited references per year. As shown in Fig. 3, the resulting network contains 924 nodes and 2593 links. A modularity Q of 0.8803 indicates well-defined clusters, while an average silhouette score of 0.5694 reflects moderate cluster homogeneity^20,21^.

In Fig. 3, node size reflects citation frequency and highlights seminal publications that underpin BM research. The most influential works, such as Shim^31^, Srikanth^32^ and Wan^33^, identified by the network are summarized in Table 2, which provides a concise view of the field’s core knowledge base.

**Table 2:** The ten most cited publications in the discipline of bridge management.

No	References	Title	Published in
1	^32^	Deterioration models for prediction of remaining useful life of timber and concrete bridges: A review	Journal of Traffic and Transportation Engineering
2	^31^	Development of a bridge maintenance system for prestressed concrete bridges using 3D digital twin model	Structure and Infrastructure Engineering
3	^33^	Development of a Bridge Management System Based on the Building Information Modeling Technology	Sustainability
4	^35^	Bridge condition rating data modeling using deep learning algorithm	Structure and Infrastructure Engineering
5	^39^	Bridge life-cycle performance and cost: analysis, prediction, optimisation and decision-making	Structure and Infrastructure Engineering
6	^40^	Maintenance Cost Optimization for Bridge Structures Using System Reliability Analysis and Genetic Algorithms	Journal of Construction Engineering and Management
7	^34^	Developing A Semi-Markov Process Model for Bridge Deterioration Prediction in Shanghai	Sustainability
8	^38^	Ontologies-based Domain Knowledge Modeling and Heterogeneous Sensor Data Integration for Bridge Health Monitoring Systems	IEEE Transactions on Industrial Informatics
9	^37^	Building Information Modeling (BIM) for transportation infrastructure	Automation in Construction
10	^36^	Bridge maintenance prioritization using analytic hierarchy process and fusion tables	Automation in Construction

Table 2 summarizes the ten most cited publications, which collectively emphasize four recurring research strands: (1) deterioration and remaining-life modelling^32^; (2) maintenance and intervention optimization under budget and performance constraints^34^; (3) condition assessment and defect detection using data-driven methods (e.g., machine learning)^35^; and (4) decision-support tools for network-level management^36^. These highly cited studies form the methodological and application backbone of BM research.

Emerging technologies such as Building Information Modeling (BIM), Digital Twins (DT), and Structural Health Monitoring (SHM) have garnered increasing attention in recent BM studies. Wan et al.^33^ introduced a BIM-based BMS with standardized modeling and coding rules tailored to China’s bridge industry, enhancing BM efficiency. Costin et al.^37^ manually reviewed 189 BIM-related studies in infrastructure but lacked scientometric analysis. Shim et al.^31^ proposed a DT-based BMS integrating key data across the bridge lifecycle. Li et al.^38^ developed an SHM ontology model using semantic web technologies to improve information sharing. In addition, Frangopol et al.^39^ presented a life-cycle BM framework integrating utility theory, risk attitudes, and SHM optimization. Ghodoosi et al.^40^ further proposed a cost-effective intervention model balancing safety and life-cycle cost. Collectively, these studies offer diverse technologies and frameworks aimed at advancing BM under various contexts.

### Cluster identification

**Fig. 4:**
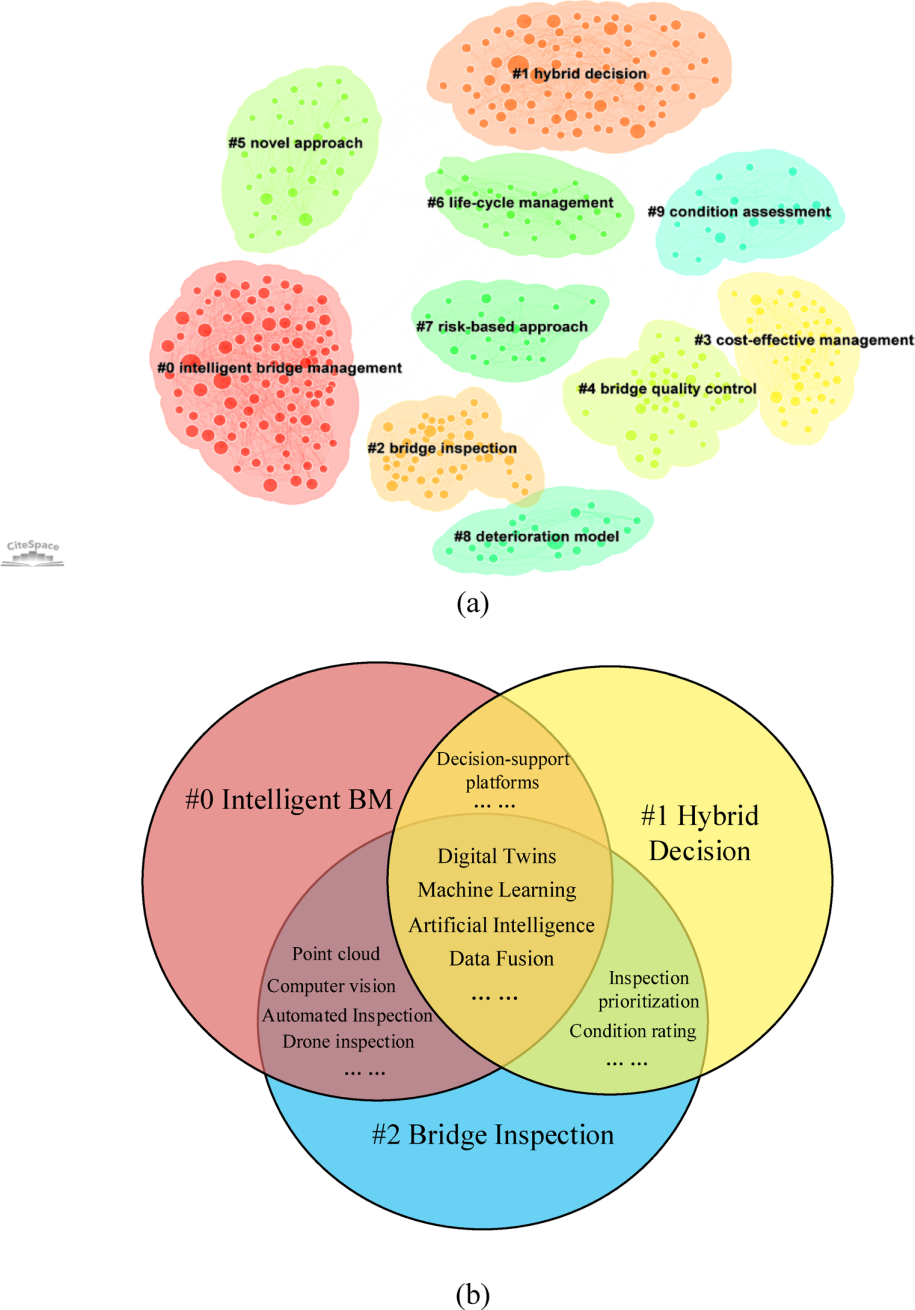
Knowledge-domain clustering and interpretive cross-pollination analysis in BM research: (**a**) CiteSpace-derived cluster map; (**b**) conceptual synthesis of the shared and distinctive themes among Cluster #0, Cluster #1, and Cluster #2.

As the second analytical step, BM publication clusters were examined to identify knowledge system outliers. CiteSpace generates candidate cluster labels from titles, keywords, and abstracts using log-likelihood ratio (LLR), mutual information (MI), and latent semantic indexing (LSI)^20,25^. In this study, all three outputs were inspected during interpretation; however, the labels reported in Fig. 4a are the LSI-derived labels based on abstract terms because they provided the most stable, concise, and substantively interpretable summaries at the cluster level. LLR and MI outputs were used as diagnostic and comparative checks^26^ rather than as the final displayed labels. Representative papers discussed for each cluster were selected on the basis of citation prominence within the cluster together with substantive relevance to the thematic label under discussion.

Figure 4a reports LSI-derived cluster labels extracted from abstract terms, with label size proportional to publication count. Figure 4b is a manual interpretive synthesis developed from the recurring themes, representative studies, and functional emphases identified within Clusters #0, #1, and #2. The clustering result should not be interpreted as a set of fully isolated research silos. Rather, it reveals several adjacent thematic streams within BM that share enabling technologies but differ in their primary analytical function. In particular, Clusters #0, #1, and #2 collectively reflect the field’s broader digital transformation, while remaining distinguishable in emphasis: Cluster #0 centres on intelligent management architectures and system integration, Cluster #1 on decision-support logic and prioritisation, and Cluster #2 on inspection-oriented data acquisition and condition interpretation. To complement the automated LSI-based labeling, Fig. 4b provides a manual interpretive “cluster cross-pollination” synthesis that visualises the shared and distinctive elements among these three clusters. In this sense, the overlap among Clusters #0, #1, and #2 should be understood as functional complementarity rather than conceptual redundancy. Clusters are indexed by size (#0 largest; #9 smallest), and all show strong clustering quality (silhouette > 0.86)^21^. This cluster-level cohesion does not contradict the lower average silhouette score (0.5694) reported for the overall co-citation network in Fig. 3, because the latter is a global network-level indicator, whereas the values reported here refer to the internal cohesion of individual clusters after partitioning.

More specifically, Fig. 4b indicates that the interaction among Clusters #0, #1, and #2 follows a structured cross-pollination pattern rather than a simple thematic overlap. The central intersection contains enabling technologies such as digital twins, machine learning, artificial intelligence, and data fusion, suggesting that these methods serve as common technical foundations across intelligent BM, hybrid decision-making, and bridge inspection. The partial overlap between Clusters #0 and #1 reflects the convergence of intelligent management architectures and decision-support functions, whereas that between Clusters #0 and #2 captures inspection intelligence and automated condition-data acquisition, including point-cloud processing, computer vision, automated inspection, and drone-assisted inspection. The overlap between Clusters #1 and #2 further shows how inspection-derived evidence is translated into decision variables through condition rating and inspection prioritization. Therefore, the three clusters should be interpreted as functionally interconnected components of an emerging data–model–decision ecosystem in BM, rather than as isolated silos or a single undifferentiated digital-transformation cluster.

Cluster #0 (“Intelligent Bridge Management”) is the largest (96 articles; silhouette = 0.965) and concentrates on AI-enabled BM, including smart systems and digital infrastructures such as BIM^37^, digital twins^31^, BMS^33^, and intelligent SHM^8^. The dominance of recent outputs (42 articles in 2021–2023) indicates that these topics are at the current research frontier. Cluster #1 (“Hybrid Decision”) targets decision-making, a core BM function, and emphasizes advanced decision-support methods and systems. Given the complexity and heterogeneity of BM data, bridge agencies increasingly rely on computerized decision-support platforms^14^. Representative studies in this cluster embed condition prediction, condition evaluation, and prioritization logic into intervention planning and resource allocation^34,36^.

A key complementary contribution is the GIS–AHP framework by Contreras-Nieto et al.^36^, which incorporates expert weighting of bridge condition and average daily traffic (ADT) information for BM decision support. Cluster #2 focuses on inspection innovations, exemplified by computer-vision-based defect detection^41^ and NLP-enabled automation of inspection and condition prediction^42^. These methods respond to escalating preservation demands under aging infrastructure and constrained maintenance, rehabilitation, and repair (MR&R) resources^43^, underscoring the operational value of timely inspection and preventive maintenance. Clusters #3 and #4 further cover cost-effective management and quality assurance. They include BM optimization under fiscal constraints^44^, network-level maintenance scheduling and post-disaster prioritization^45,46^, and sustainability-oriented quality control that integrates technical and non-technical indicators^47^, with additional support from studies linking BMS and SHM for performance monitoring and control^48^. Beyond these, other major clusters align with established BM domains, including life-cycle management^49^, risk management^50^, deterioration modeling^51^, and condition assessment^35^.

### Keyword co-occurrence network analysis

**Fig. 5:**
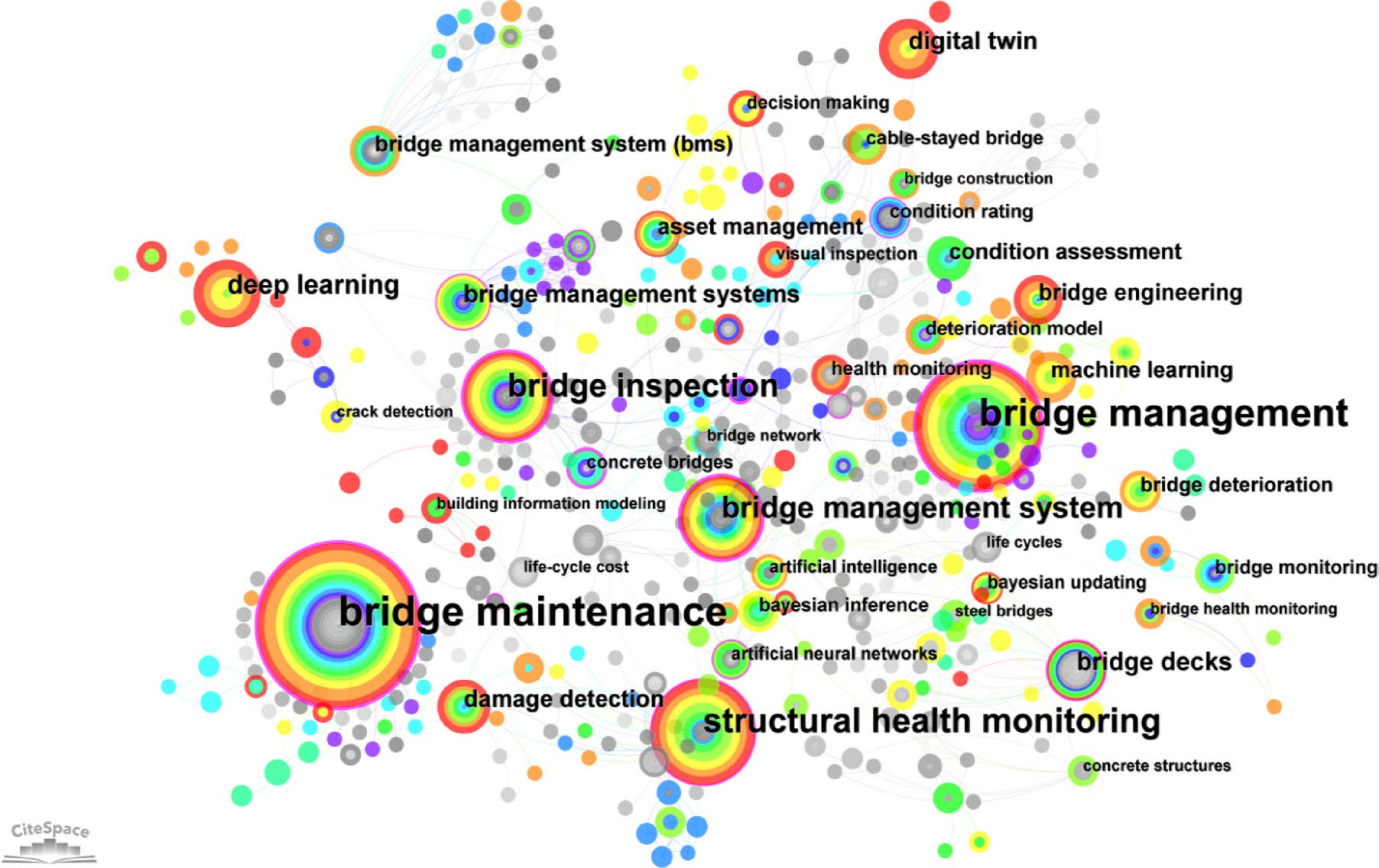
Keywords co-occurrence network.

As shown in Fig. 5, “bridge maintenance” is the most frequent keyword (occurrence frequency: 146). Although it is often conflated with bridge management (BM), maintenance represents only one component within the broader BM framework^10^, which also covers cost, safety, sustainability, and structural integrity^52^. Consistent with this relationship, “bridge management” ranks second (102), indicating that maintenance-oriented studies remain an important knowledge base while the field increasingly adopts a more comprehensive BM perspective.

Beyond these foundational terms, the network highlights core enabling themes. Structural Health Monitoring (SHM) supports real-time data acquisition for BM decisions and is widely recognized for improving management efficiency and decision quality^9,53,54^. Bridge Management Systems (BMS) (52) function as integrative platforms for network-level management, supporting long-term safety^4^ and cost efficiency^14^. Bridge inspection also emerges as a persistent focus because it underpins maintenance planning and network reliability^55^. In parallel, the rise of AI and digital technologies is evident in the prominence of terms such as deep learning, machine learning, and digital twin, reflecting their growing influence on BM theory and practice^9,50,56,57^.

### Co-authorship analysis

**Fig. 6:**
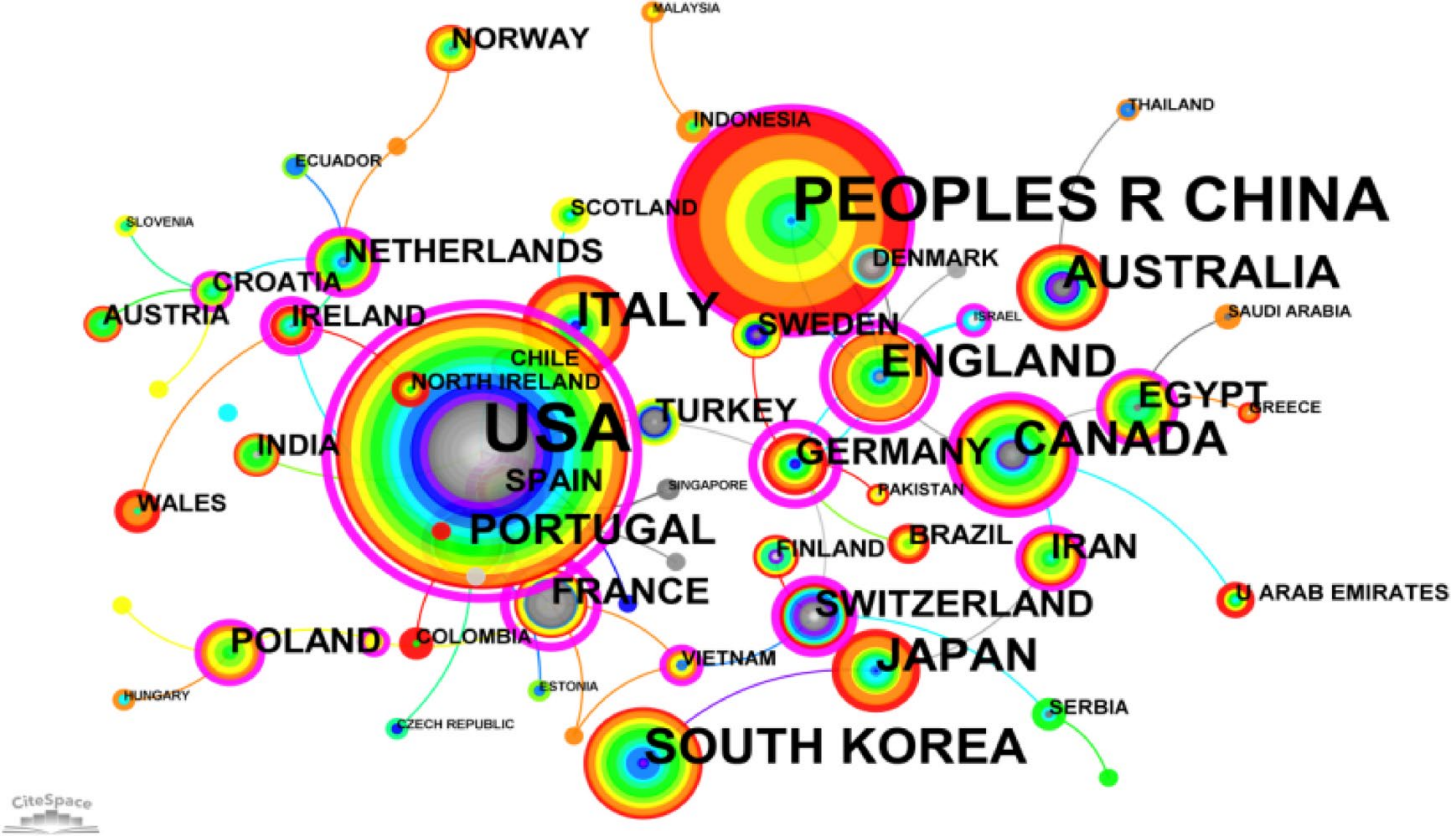
Collaboration network of countries.

The country co-authorship network (Fig. 6) comprises 63 nodes and 148 links, reflecting active international collaboration in BM research. In terms of publication output, the USA, China, South Korea, Italy, and Canada are the leading contributors, indicating their central roles in global knowledge production. Detailed statistics for the top ten countries, including frequency and centrality, are reported in Table 3. By betweenness centrality^58^, the USA, France, Portugal, England, and Canada rank highest, suggesting a stronger bridging function in cross-national knowledge exchange.

**Table 3:** Top 10 countries of co-authorship network.

No	Countries	Count	Centrality
1	USA	306	0.91
2	CHINA	216	0.06
3	South Korea	63	0.04
4	Italy	54	0.06
5	Canada	54	0.39
6	England	45	0.65
7	Australia	44	0.06
8	Japan	42	0.06
9	Portugal	32	0.74
10	France	19	0.97

**Fig. 7:**
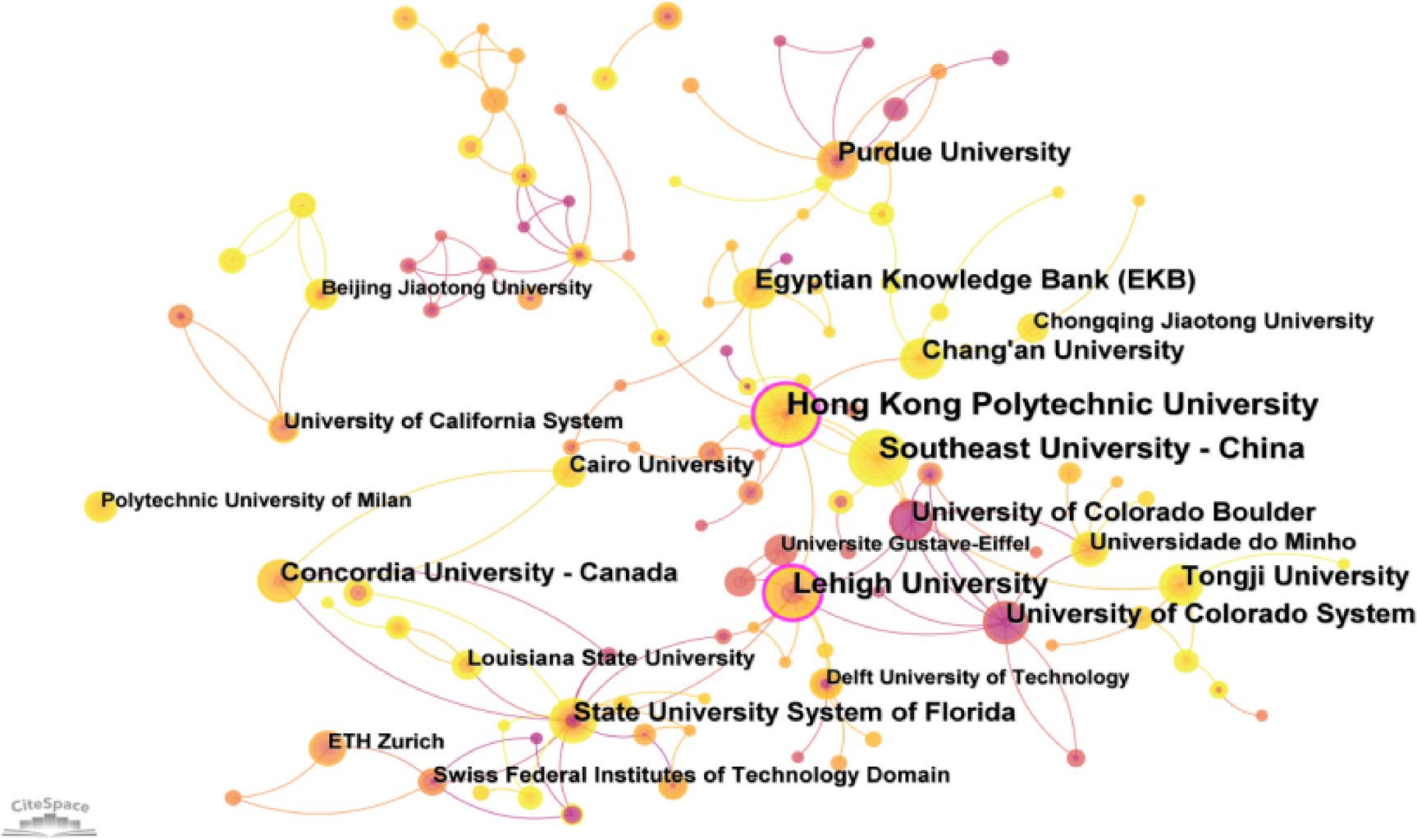
Collaboration network of institutes.

The institutional collaboration network generated by CiteSpace contains 415 nodes and 393 links (Fig. 7). Several institutions—such as The Hong Kong Polytechnic University, Lehigh University, the State University System of Florida, Southeast University, and the University of Colorado Boulder—exhibit relatively dense connections, forming a major collaborative consortium. In contrast, some institutions (e.g., Purdue University, Beijing Jiaotong University, and Concordia University) appear in comparatively smaller subnetworks. As summarized in Table 4, the top ten institutes have constituted the main institutional backbone of BM research over time: The Hong Kong Polytechnic University, Southeast University, and Lehigh University lead in publication volume (each exceeding 20 articles), while The Hong Kong Polytechnic University, Lehigh University, and the State University System of Florida show higher centrality, indicating broader influence within the collaboration structure.

**Table 4:** Top 10 institutes of co-authorship network.

No	Institutes	Count	Centrality
1	Hong Kong Polytechnic University	30	0.16
2	Southeast University	29	0.01
3	Lehigh University	25	0.13
4	University of Colorado System	19	0.02
5	Tongji University	18	0.03
6	Egyptian Knowledge Bank	18	0.05
7	University of Colorado Boulder	17	0.02
8	Purdue University	16	0.01
9	Chang’an University	16	0.03
10	State University System of Florida	16	0.07

**Fig. 8:**
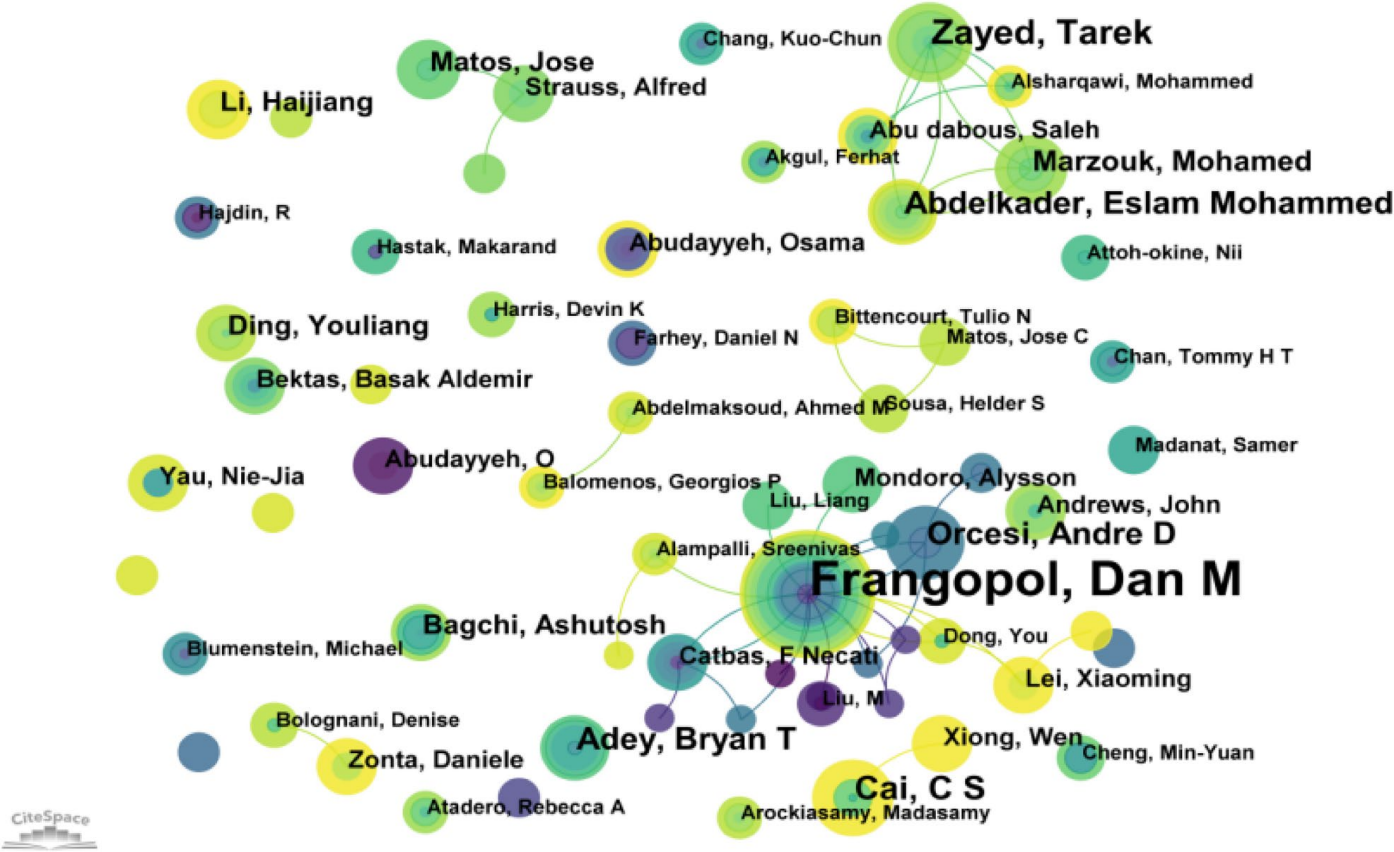
Map of the BM author collaboration network.

The author collaboration network (Fig. 8) includes 506 nodes and 317 links, indicating a field organised around several collaboration communities rather than a single cohesive group. A prominent community is centred on Dan M. Frangopol and frequent collaborators, while another community is led by Tarek Zayed; additional smaller groups (e.g., those associated with Matos, Jose and Cai, C. S.) further contribute to the diversity of BM research. Table 5 lists the top ten authors by publication count, with Dan M. Frangopol ranked first, followed by Tarek Zayed, Cai, C. S., Mohammed Z. E. B., and Orcesi, Andre D. The leading authors are geographically distributed; among the top ten, two are affiliated with institutions in the USA, while the others are based in different countries, reflecting the internationalised authorship structure of BM research.

**Table 5:** Top 10 authors of collaboration network.

No	Author	Institution	Country	Count
1	Dan M. Frangopol	Lehigh University	USA	29
2	Tarek Zayed	Concordia University	Canada	9
3	Cai, C S	Louisiana State University	USA	8
4	Mohammed Z. E. B	University of Cambridge	England	8
5	Orcesi, Andre D	University of Gustave Eiffel	France	7
6	Adey, Bryan T	ETH Zurich	Switzerland	7
7	Marzouk, Mohamed	Cairo University	Egypt	7
8	Bagchi, Ashutosh	Concordia University	Canada	6
9	Matos, Jose	Minho University	Portugal	5
10	Ding, Youliang	Southeast University	China	5

Scientific implications of co-authorship analysis can be gained as follows. The collaboration networks (Figs. 6 and 7) and isible hubs, with dense intra-hub ties but comparatively weaker cross-regional connectivity. This structure facilitates rapid method diffusion within leading groups, yet may slow translation and benchmarking across jurisdictions with heterogeneous data standards, inspection regimes, and governance contexts. These patterns reinforce the need for interoperable data/ontology standards and shared evaluation protocols so that emerging AI/DT/SHM-enabled approaches can be replicated and compared across regions rather than remaining fragmented case-specific solutions.

### Evolution trends of BM knowledge

Citation bursts highlight rapidly emerging research trends. Supplementary Table S3 lists the top 24 references [9, 31–35, 39–44, 62–75] with the strongest bursts, beginning in 2003, a period aligned with early BM development. From 2003 to 2009, research centered on life-cycle management, particularly the relationship between cost and reliability^59–61^. Among the top bursts, life-cycle management and SHM rank second and third in intensity^39,59^.

Since 2010, focus has shifted toward digitalization and integration of BM with technologies like BIM and DT, with BMS increasingly adopted in practice^37^. Bridge maintenance became a key theme in 2019, emphasizing preventive strategies, though corrective approaches are also emerging^10^. The most intense burst involves DT-based preventive systems^31^. From 2017 onward, BM research has increasingly adopted intelligent methods such as machine learning^62^, deep learning^35^, and ontology-based frameworks^38^.

**Fig. 9:**
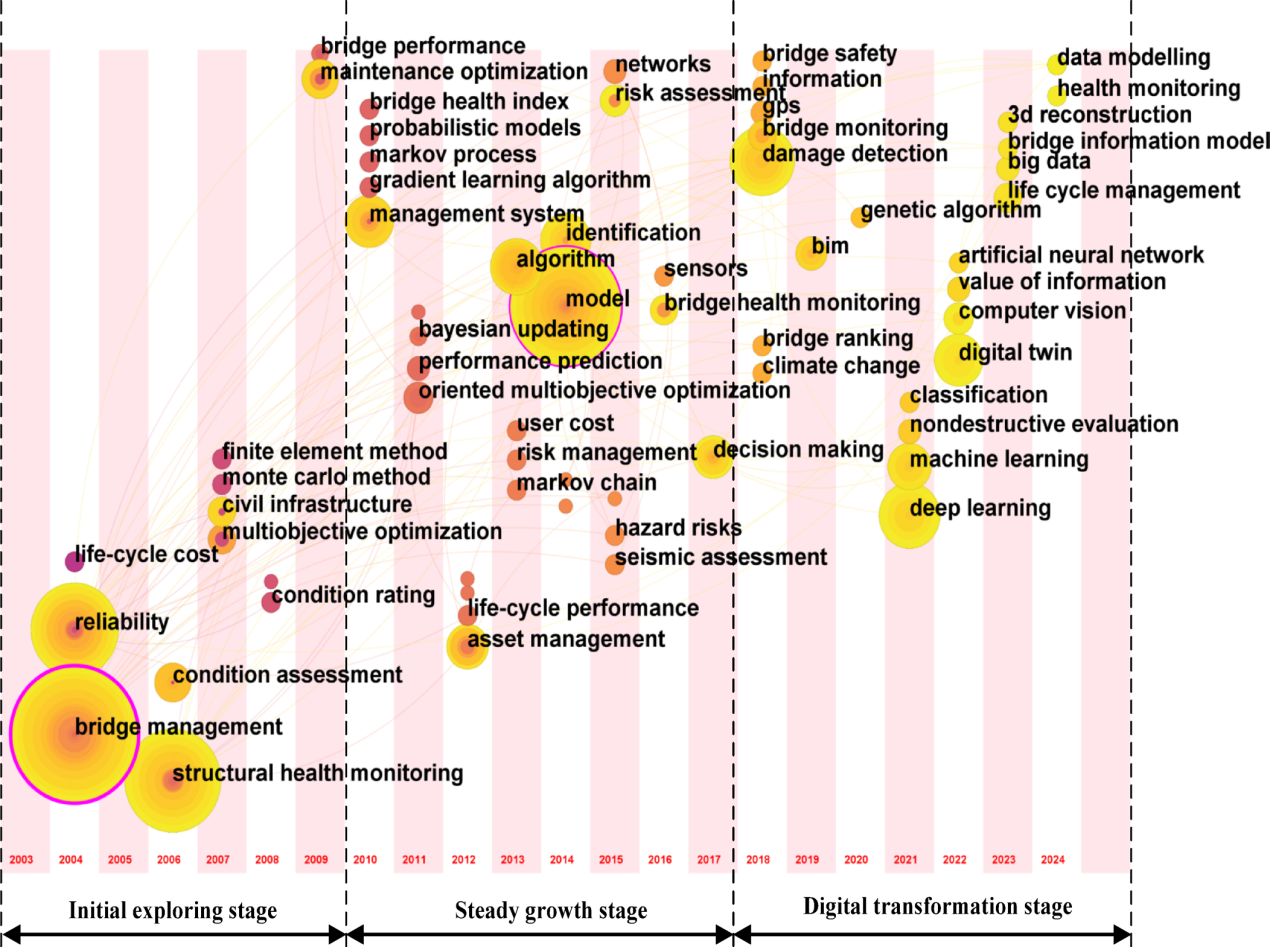
A time zone view of keywords.

Figure 9 visualizes keyword evolution through a time-zone view and clarifies not only when new topics emerged, but also how the field shifted from maintenance-oriented reliability studies to digitally enabled and intelligence-driven BM. In the exploratory stage, the field focused on balancing reliability and life-cycle costs, with foundational topics such as BM, SHM, and condition assessment. The steady growth stage introduced digitalization, reflected in terms such as model, system, and algorithm, together with asset and risk management. In the digital transformation stage, intelligent technologies—including deep learning, machine learning, digital twins, and computer vision—became prominent, indicating a clear transition from conventional management routines toward predictive, data-intensive, and integrative BM approaches.

Taken together, the findings in this section provide a descriptive, evidence-based overview of BM’s publication growth, intellectual clusters, thematic hotspots, collaboration structure, and temporal evolution (Figs. 2, 3, 4, 5, 6, 7, 8 and 9; Tables 2, 3, 4 and 5; Table S3). Building on these results, the following section focuses on synthesis and interpretation rather than descriptive statistics, and the subsequent section distills evidence-informed research gaps and future directions.

For clarity, the five items below are interpreted as evidence-informed underdeveloped or weakly integrated directions rather than as definitive absences in the literature.

## Discussion

### Mapping the intellectual structure of BM research

**Fig. 10:**
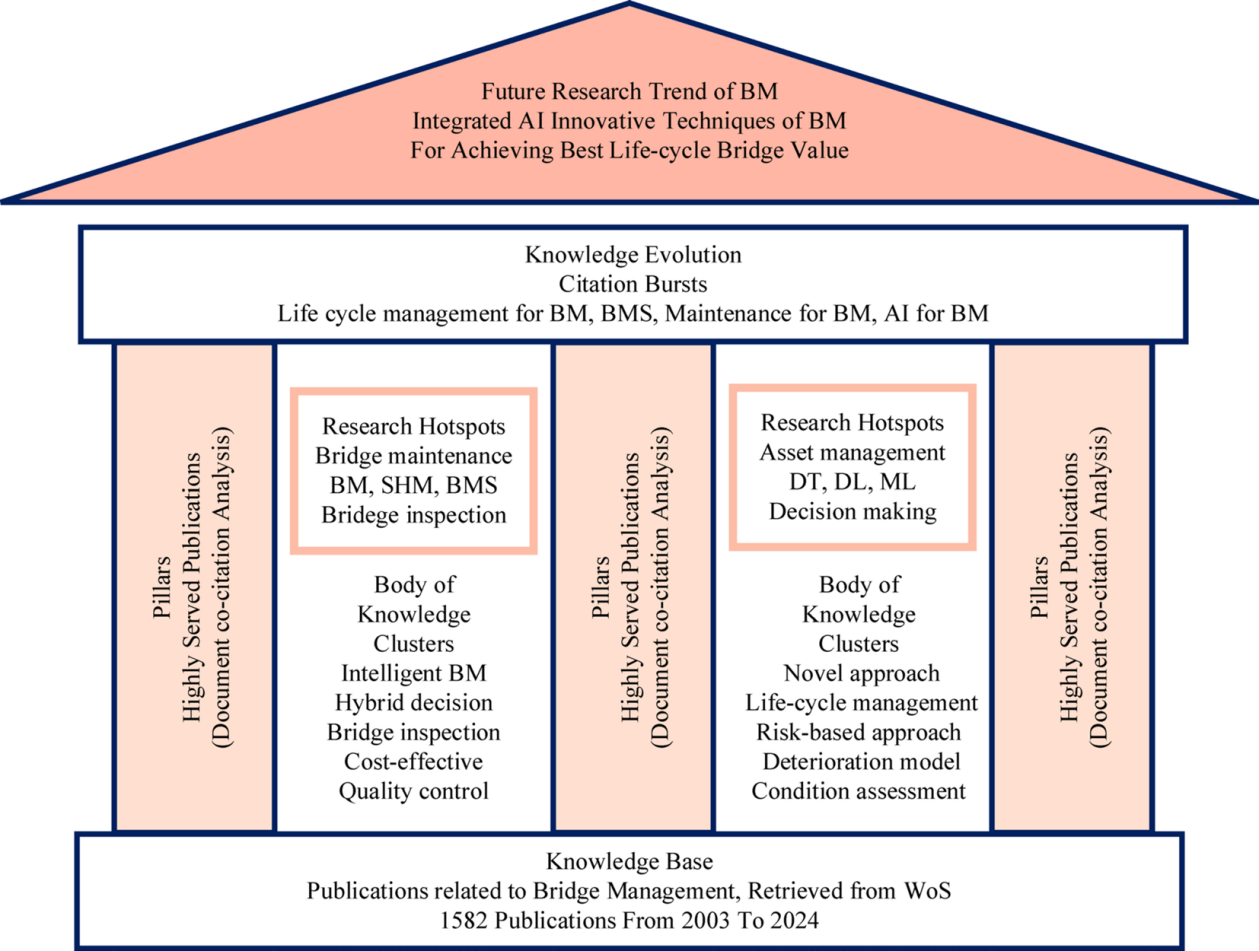
BM knowledge map.

Building on the results reported in the Findings section (Figs. 2, 3, 4, 5, 6, 7, 8 and 9; Tables 2, 3, 4 and 5; Table S3), we interpret how BM research has consolidated around identifiable intellectual bases, thematic streams, and evolving priorities, while highlighting cross-cluster linkages that are not immediately visible from descriptive statistics alone^4,6^. Figure 10 integrates these patterns into a six-part knowledge map (knowledge base, influential publications, clusters, hotspots, thematic evolution, and strategic directions). In addition, the collaboration analyses (Figs. 6, 7 and 8; Tables 3, 4 and 5) indicate that knowledge diffusion is shaped by a small number of highly connected countries and institutions, whereas cross-community author links are comparatively sparse—helping explain why some themes progress in parallel rather than converging. Together, these insights provide context for the targeted research gaps outlined next.

At the same time, this global knowledge map should not be interpreted as a geographically neutral representation of BM practice. Because the most visible publication hubs are concentrated in countries and institutions with stronger research infrastructure, indexing visibility, and digital capacity, the map is more reflective of high-income and technology-intensive BM research than of the full diversity of global bridge-management conditions. This imbalance is especially relevant to emerging hotspots such as digital twins, AI, and advanced SHM, which remain less visible in lower-resource and Global South contexts where bridge governance is more strongly shaped by inspection capacity, funding constraints, local standards, and practical maintenance needs. Accordingly, the scientometric patterns reported here should be read both as a map of BM research development and as an indication of the field’s current digital divide.

Scientifically, Fig. 10 indicates a converging BM research logic from data acquisition and representation (inspection, SHM, BIM/DT) to inference (deterioration, reliability, AI) and then to portfolio governance (prioritisation, sustainability, risk/resilience). This means that the BM literature is no longer evolving as a set of isolated technical topics; rather, it is progressively forming a connected data–model–decision pipeline. The five gaps identified below can therefore be interpreted as missing links across this pipeline, especially regarding interoperability, uncertainty handling, and deployable BMS-enabled workflows.

This knowledge map consolidates fragmented insights into a transparent and updateable synthesis, making cross-theme linkages explicit for evidence-informed interpretation and agenda setting. At this point, the study responds to *RQ*1 and *RQ*2, providing both a structured view of BM’s evolution and a basis for identifying future research directions.

### Research gaps and future directions

To identify research gaps (RQ3), we triangulated three sources of evidence: (1) scientometric signals from co-citation clustering, keyword co-occurrence, timeline evolution, and citation bursts (Figs. 3, 4, 5 and 9; Table S3); (2) a keyword-coverage check on WoS metadata (Supplementary Table S2); and (3) expert validation by the Delphi panel (Supplementary Table S1). Importantly, no single bibliometric indicator was used to define a gap on its own. Instead, each proposed gap was inferred from a combination of thematic prominence, cross-cluster discontinuity, temporal evolution, and relative underrepresentation in keyword coverage, and was then cross-checked through expert interpretation. To make this logic explicit, Supplementary Table S4 presents a gap-mapping matrix linking the main bibliometric observations to the corresponding evidence-informed future directions. In addition, representative highly cited and recent papers within the relevant clusters were manually examined to judge whether a theme was merely low-frequency or instead substantively underdeveloped, fragmented, or weakly integrated into the broader BM workflow.

To further contextualize the five persistent gaps with quantitative evidence, we conducted a term-based coverage analysis using CiteSpace-exported WoS keyword metadata. For each gap theme, a representative, case-insensitive term set was identified and matched against metadata and keyword hits across the full dataset (N = 1582). The results show that themes related to IT-enabled BM/BMS integration and inspection planning have the highest coverage (both n = 133; 8.41%), followed by hybrid decision-making (n = 96; 6.07%). In contrast, sustainability/LCA/LCSA (n = 40; 2.53%) and risk & resilience (n = 57; 3.60%) remain comparatively underrepresented, providing one quantitative input to the evidence-informed interpretation presented below.

**Fig. 11:**
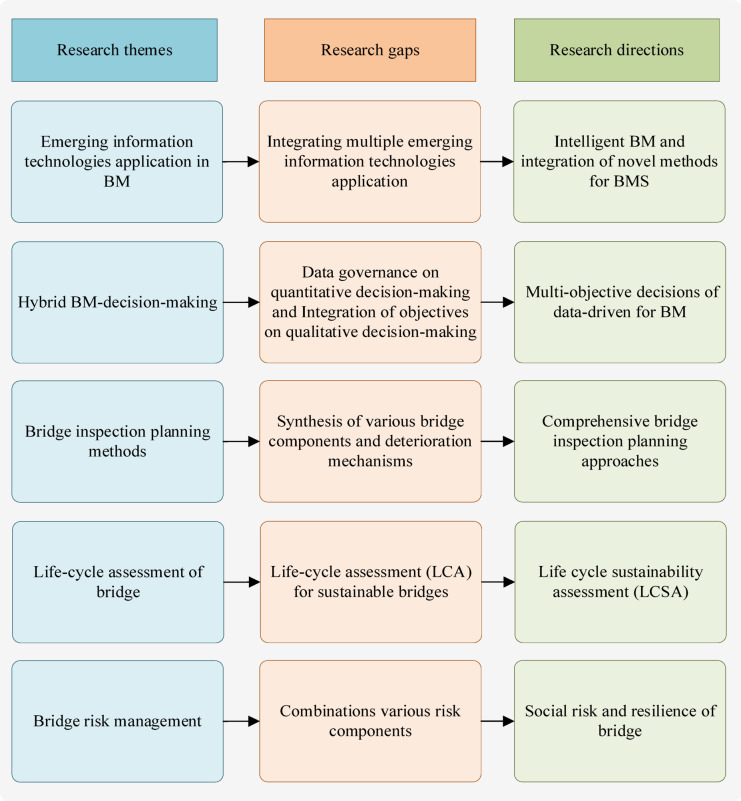
Research route linking research gaps to future research directions.

Supplementary Table S4 makes explicit how the proposed gaps were inferred from combined bibliometric observations rather than from any single cluster, burst, or keyword count. Figure 11 then synthesises these mapped relationships into an integrated research-route diagram.

#### Gap 1: emerging information technologies application in BM

Recent BM studies increasingly emphasise emerging information technologies—particularly AI-enabled analytics, BIM, digital twins, and integrated BMS/SHM platforms—concentrated in the “Intelligent Bridge Management” cluster and related streams (Fig. 4). These technologies are commonly positioned to improve network-level visibility and decision timeliness through data-driven condition assessment and predictive maintenance, leveraging BMS architectures^14,63^, SHM/AI pipelines^9,62^, and digital-twin concepts^64^. Nevertheless, much of the literature remains focused on isolated technical demonstrations, with limited discussion of end-to-end integration (data governance, interoperability, and organisational adoption) needed for routine BM deployment^6^. Future research should therefore develop interoperable, life-cycle-oriented BM architectures that connect sensing, modelling, and decision support in a consistent workflow, supported by standardized data schemas, quality control, cybersecurity, and workforce readiness for large-scale implementation. Particular attention should be paid to adaptable and modular deployment pathways for lower-resource regions, where digital capacity, institutional standards, and inspection regimes may differ substantially from those represented in the dominant BM literature.

#### Gap 2: hybrid decision-making of BM

BM decision-making is increasingly multi-criteria and data-intensive^65^, yet prevailing approaches are dominated by quantitative optimisation and prediction models, with limited integration of qualitative evidence such as expert judgement, stakeholder values, and governance constraints^66^. Delphi feedback further indicates that data governance (collection, storage, and interoperability) is a practical bottleneck for hybrid decision frameworks. Research is needed on hybrid decision-support platforms that combine quantitative models with structured qualitative inputs (e.g., risk tolerance, service-disruption consequences, and equity considerations) and that operate across time horizons from emergency actions to long-term investment planning. Supplementary Table S2 (n = 96; 6.07%) suggests that, although decision-analytic methods are active, genuinely hybrid frameworks remain comparatively underdeveloped.

#### Gap 3: synthesis of various bridge inspection planning methods

Inspection-related studies have expanded rapidly (Figs. 5 and 9), including computer-vision, robotics, and drone-enabled approaches, but many target single components or failure modes^67^ and are rarely embedded in network-wide, deterioration-aware inspection planning^55^. As a result, the literature provides limited guidance on how to combine multi-source inspection data to optimise inspection type, timing, and monitoring intensity across bridge portfolios. Related AEC studies have also demonstrated the potential of deep-learning-based instance segmentation using BIM-generated synthetic point clouds for automated object-level point-cloud interpretation^68^, although such approaches have not yet been systematically translated into bridge inspection planning workflows. Future work should develop risk- and deterioration-informed inspection planning frameworks that integrate heterogeneous data streams (images, signals, and environmental/corrosion indicators) into a unified scheduling logic and allow adaptive reprioritisation after extreme events. Although inspection keywords are well represented (Supplementary Table S2: n = 133; 8.41%), cross-source fusion and network-level scheduling remain comparatively scarce.

#### Gap 4: life-cycle assessment for sustainable bridges

While sustainability is increasingly discussed in BM-related clusters, bridges remain underrepresented in life-cycle sustainability applications compared with other built assets^69^. This is also reflected in the low coverage of sustainability/LCA/LCSA terms in our dataset (Supplementary Table S2: n = 40; 2.53%), suggesting that sustainability is not yet routinely operationalised in BM decisions. Building on recent review discussions^49^, BM-specific LCSA frameworks are needed to support early-stage planning and explicit trade-offs across environmental, economic, and social dimensions.

#### Gap 5: bridge risk and its resilience

Risk-related research appears in Cluster #7, but it remains relatively limited in the overall BM corpus (Supplementary Table S2: n = 57; 3.60%). Existing approaches often focus on single hazards and can be resource-intensive, limiting scalability for network-level decision support^70^. Accordingly, the resilience gap identified here is not inferred from citation bursts alone; rather, it emerges from the combined evidence of limited keyword coverage, relatively narrow cluster representation, and weak integration with the broader BM data-model-decision pipeline. This indicates a need to couple deterioration processes with multi-hazard exposure and system-level consequences in a tractable modelling framework.

Future studies should advance probabilistic, multi-risk and resilience models that capture compounding effects and uncertainty, and that represent social impacts such as service disruption and equity. Integrating resilience metrics with network analysis and life-cycle evaluation, supported by data-driven updating, would enable proactive intervention prioritisation rather than post-disaster reaction.

Overall, these gaps outline a coherent research agenda: interoperable digital infrastructures for BM, genuinely hybrid decision models, portfolio-level inspection planning with data fusion, BM-tailored LCSA for sustainability trade-offs, and scalable multi-risk resilience modelling. Figure 11 should therefore be read as an evidence-integration map: the left side condenses the main scientometric signals, the middle layer identifies the corresponding underdeveloped themes, and the right side translates them into future research directions. In this sense, the figure does not merely restate the gaps visually; it explains how quantitative patterns and expert cross-checking jointly support the proposed agenda.

## Conclusion and implications

This study presents an integrated scientometric review of bridge management (BM) based on 1,582 Web of Science Core Collection journal articles (2003–2024), combining PRISMA screening, CiteSpace mapping, and Delphi-based keyword refinement. The results delineate three developmental stages, identify ten major co-citation clusters and emerging technology hotspots, and reveal an internationally connected yet uneven collaboration structure, synthesized into a BM knowledge map.

The findings offer academic and practical implications. For researchers, the knowledge map supports positioning new studies and points to five priorities: interoperable digital BM architectures, rigorously validated hybrid decision models, network-level inspection planning enabled by multi-source data fusion, BM-tailored life-cycle sustainability assessment, and scalable multi-hazard resilience modeling. For agencies and policymakers, particularly in developing regions where data and resources are constrained, the identified evidence base and collaboration hubs can support benchmarking, capacity building, and implementation planning. Continued progress in BM will depend on interdisciplinary collaboration across civil engineering, sensing, AI, and public policy to translate methods into deployable systems.

Several limitations should be noted. First, the dataset was restricted to the Web of Science Core Collection and English-language journal articles, which may under-represent regional outputs, non-English scholarship, conference proceedings, and agency or industry reports. This restriction may also introduce database bias by favoring topics, institutions, and regions with stronger visibility in WoS indexing. As a result, the knowledge map is likely to capture high-income and technology-visible research more strongly than BM practices and challenges documented in lower-resource or Global South contexts. Second, the Delphi panel was intentionally small and senior; although selection criteria and consensus rules were pre-defined, expert judgment remains context-dependent. Third, term-based gap quantification provides indicative evidence but does not fully capture study-level engagement with each theme. Future work can triangulate multiple databases, broaden stakeholder panels, and combine scientometric mapping with systematic content analysis.

## Supplementary Information

Below is the link to the electronic supplementary material.

Supplementary Material 1

## Data Availability

Due to licensing restrictions, the bibliometric dataset retrieved from Web of Science cannot be publicly shared, but is available from the authors on reasonable request.
