# Supplementary material for: Exploring a comprehensive knowledge map for bridge management research: a Delphi-enhanced scientometric analysis
Source: Sci Rep. 2026 May 7;16:21078. doi: 10.1038/s41598-026-51462-6 (PMC13341768; doi:10.1038/s41598-026-51462-6)
Supplement: Supplementary file 1 — Supplementary Material 1 [file 41598_2026_51462_MOESM1_ESM.pdf]

Supplementary Information

Title: Exploring a comprehensive knowledge map for bridge management research: a Delphi-enhanced scientometric analysis

Authors: Peng Peng, Zuocai Wang, Peng Cui, Hongzhe Yue, Junfeng Yao, Sainan Lyu

Submission ID: 08df7659-3512-4a94-a594-fd0f619fe0ac

Table S1. Composition of the Delphi panel and expert background characteristics.

Panel member (coded)	Age (years)	Highest degree attained	Current position/role	Professional experience (years)
E1	61	Bachelor	Senior engineer	36
E2	58	Master	Senior engineer	34
E3	56	Master	Professor	33
E4	52	Master	Professor	27
E5	49	Doctor	Professor	22

Notes: Experts are anonymised (E1-E5) to protect privacy. Eligibility criteria were predefined as: (i) ≥20 years of research or professional experience related to bridge management (BM) (e.g., inspection, maintenance, condition assessment, deterioration modelling, intervention planning, asset management); (ii) demonstrated domain expertise evidenced by peer-reviewed publications and/or leadership roles in BM-related projects; (iii) familiarity with emerging BM-related digital technologies (e.g., SHM, BIM, digital twins) and their practical applications; and (iv) willingness to participate in all iterative rounds. Panel composition: three academics (professors) and two practitioners (senior engineers); age 49-61 years; BM-related professional experience 22-36 years. All panel members participated in all Delphi rounds.

Table S2. Term-based coverage of representative themes corresponding to the five research gaps.

Gap theme	Representative term set (case-insensitive)	Keyword hits (n)	Share of total (%)
Gap 1: IT-enabled BM/BMS integration	digital twin; BIM; building/bridge information modeling; deep learning; machine learning; artificial intelligence; computer vision; ontology; information extraction; point cloud/3D reconstruction; unmanned aerial vehicle	133	8.41
Gap 2: Hybrid decision-making	decision making; multi-criteria decision making; AHP/analytic hierarchy process; fuzzy*; Bayesian*; Markov*; genetic algorithm*; multiobjective optimization; uncertainty; value of information	96	6.07
Gap 3: Inspection planning synthesis	bridge inspection; inspection; visual inspection; condition assessment/rating/index; maintenance planning; preventive maintenance; prioritization; bridge ranking	133	8.41
Gap 4: Sustainability/LCA/LCSA	life cycle/life-cycle; life cycle assessment; life cycle cost/costing; life cycle analysis/management; environmental impact; carbon footprint; carbon dioxide; climate change	40	2.53
Gap 5: Risk & resilience	risk; risk assessment/management/analysis; safety; fragility/fragility curves; seismic assessment; earthquake; hazard risks; multiple hazards; bridge failure(s); resilience indicator	57	3.60

Notes: Based on CiteSpace-exported WoS keywords. n counts cumulative keyword hits (N = 1,582); percentages are indicative (not unique-record shares).

Table S3. Top 24 References with the Strongest Citation Bursts

References	Year	Strength	Begin	End	2000-2024
[62]	2003	5.36	2004	2008	
[63]	2007	4.92	2010	2012	
[64]	2011	4.37	2011	2013	
[65]	2013	3.91	2015	2018	
[66]	2016	3.72	2018	2020	
[67]	2016	3.98	2019	2021	
[43]	2017	3.98	2019	2021	
[40]	2017	7.23	2020	2022	
[41]	2018	5.08	2020	2022	
[44]	2017	3.80	2020	2022	
[68]	2018	3.80	2020	2022	
[69]	2017	3.38	2020	2022	
[35]	2020	6.26	2021	2024	
[32]	2020	5.08	2021	2024	
[70]	2017	3.93	2021	2022	
[71]	2017	3.44	2021	2022	
[31]	2019	8.11	2022	2024	
[33]	2019	6.05	2022	2024	
[9]	2019	3.99	2022	2024	
[72]	2018	3.99	2022	2024	
[73]	2020	3.68	2022	2024	
[39]	2021	3.68	2022	2024	
[74]	2021	3.55	2022	2024	
[75]	2020	3.34	2022	2024	

Table S4. Gap-mapping matrix linking bibliometric evidence to evidence-informed future directions

Gap theme	Main bibliometric signals	Interpretation logic	Evidence-informed future direction
IT-enabled BM/BMS integration	Cluster #0 prominence; repeated DT/AI-related bursts; high keyword coverage (8.41%)	High visibility indicates strong technological activity, but the evidence is concentrated in fragmented demonstrations rather than end-to-end deployment workflows	Develop interoperable, life-cycle-oriented BM architectures that connect sensing, modelling, and decision support
Hybrid decision-making	Cluster #1 prominence; keyword coverage 6.07%; limited linkage between optimisation studies and governance/stakeholder dimensions	Decision-support is active, but current studies remain dominated by quantitative optimisation and insufficiently integrate qualitative and governance constraints	Develop genuinely hybrid decision-support frameworks combining quantitative analytics with expert/stakeholder inputs
Inspection planning synthesis	Strong inspection-related keywords and Cluster #2 activity; repeated monitoring/inspection terms in timeline evolution; high keyword coverage (8.41%)	Inspection is highly visible, but much of the literature remains component-specific and weakly connected to network-level scheduling and fusion logic	Build portfolio-level inspection planning frameworks integrating heterogeneous inspection data and adaptive scheduling
Sustainability/LCA/LCSA	Scattered presence across several clusters; low keyword coverage (2.53%)	Sustainability appears as a recognized topic, but remains weakly operationalised in BM-specific decision frameworks	Develop BM-tailored LCSA/LCA frameworks for explicit environmental-economic-social trade-offs
Risk & resilience	Presence in Cluster #7 and timeline evolution, but low keyword coverage (3.60%) and limited integration with broader BM pipeline	Risk/resilience is visible but not yet sufficiently integrated into scalable, multi-hazard, system-level BM models	Advance probabilistic, scalable multi-risk and resilience models linked to life-cycle and network-level decision support

Table S5. Round-by-round Delphi refinement of topic-search terms used in the Web of Science retrieval

Candidate term / term group	Round 1 status	Round 2 action	Final wording after Round 3	Median	IQR	Included in final TS query
bridge management	proposed by multiple experts	retained without revision	bridge management	5	0	Yes
bridge maintenance	proposed by multiple experts	retained without revision	bridge maintenance	5	0	Yes
bridge management system	proposed by multiple experts	retained without revision	bridge management system	5	0	Yes
bridge inspection	proposed by multiple experts	retained without revision	bridge inspection	5	1	Yes
bridge health monitoring	proposed by multiple experts	retained without revision	bridge health monitoring	4	1	Yes
bridge asset management	proposed in Round 1	retained after discussion	bridge asset management	4	1	Yes
bridge project management	proposed in Round 1	retained after discussion	bridge project management	4	1	Yes
bridge construction management	proposed in Round 1	retained after discussion	bridge construction management	4	1	Yes
bridge engineering management	proposed in Round 1	retained after discussion	bridge engineering management	4	1	Yes
bridge * management	added as umbrella wildcard expression after Round 2 consolidation	retained to capture lexical variants	bridge * management	4	1	Yes
digital twin for bridges	proposed in Round 1	merged into broader BM/BMS retrieval logic to avoid overly technology-specific bias	—	—	—	No
BIM-based bridge management	proposed in Round 1	not retained as a standalone term; considered covered by bridge management system / bridge * management	—	—	—	No

Candidate term / term group	Round 1 status	Round 2 action	Final wording after Round 3	Median	IQR	Included in final TS query
SHM-based bridge management	proposed in Round 1	merged into bridge health monitoring	—	—	—	No
bridge deterioration management	proposed in Round 1	removed as overly specific and insufficiently central to BM retrieval scope	—	—	—	No
bridge maintenance decision-making	proposed in Round 1	merged into bridge maintenance and bridge management system	—	—	—	No
intelligent bridge management	proposed in Round 1	not retained as a standalone retrieval term because of low standardization and overlap with bridge management / bridge * management	—	—	—	No
bridge life-cycle management	proposed in Round 1	discussed in Round 2; excluded from final query to avoid drift toward broader life-cycle engineering literature beyond BM focus	—	—	—	No
bridge operation and maintenance	proposed in Round 1	considered relevant but merged into bridge maintenance / bridge management	—	—	—	No
bridge decision support	proposed in Round 1	merged into bridge management system	—	—	—	No
bridge condition assessment	proposed in Round 1	excluded as a standalone term because it was treated as a core BM topic captured during screening rather than as a primary search anchor	—	—	—	No

Notes: This table documents the evolution of the topic-search vocabulary across the three Delphi rounds based on the recorded panel workflow, retained consensus rules, and the final topic-search query reported in the main text. Round 1 generated a broad candidate pool from BM research and practice. Round 2 consolidated overlapping, overly narrow, or ambiguous expressions. Round 3 retained terms judged sufficiently relevant and comprehensive for topic retrieval. Median and IQR are reported only for the final retained terms that entered the consensus-based query.
